# GC-MS Chemical Characterization and In Vitro Evaluation of Antioxidant and Toxic Effects Using *Drosophila melanogaster* Model of the Essential Oil of *Lantana montevidensis* (Spreng) Briq.

**DOI:** 10.3390/medicina55050194

**Published:** 2019-05-23

**Authors:** Maria Rayane Correia de Oliveira, Luiz Marivando Barros, Antônia Eliene Duarte, Maria Gabriely de Lima Silva, Bruno Anderson Fernandes da Silva, Anita Oliveira Brito Pereira Bezerra, Cícera Datiane Morais Oliveira Tintino, Victor Afonso Pereira de Oliveira, Aline Augusti Boligon, Jean Paul Kamdem, Henrique Douglas Melo Coutinho, Irwin Rose Alencar de Menezes

**Affiliations:** 1 Laboratory of Pharmacology and Molecular Chemistry (LFQM), Department of Biological Chemistry, Regional University of Cariri—URCA, Crato-Ceará CEP 63105-000, Brazil; rayaneoliveirabio@gmail.com (M.R.C.d.O.); gabriellyscience@gmail.com (M.G.d.L.S.); brunoskarllet2@gmail.com (B.A.F.d.S.); anitaoliveira24@yahoo.com.br (A.O.B.P.B.); datianemorais@gmail.com (C.D.M.O.T.); victor.apcrato@gmail.com (V.A.P.d.O.); 2 Center for Biological and Health Sciences-CCBS, Department of Biological Chemistry Sciences, Regional University of Cariri—URCA, Pimenta, Crato-Ceará CEP 63105-000, Brazil; marivando.barros@urca.br (L.M.B.); duarte105@yahoo.com.br (A.E.D.); kamdemjeanpaul2005@yahoo.fr (J.P.K.); 3 Laboratory of Phytochemistry, Department of Industrial Pharmacy, Federal University of Santa Maria, Santa Maria RS 97105-900, Brazil; alineboligon@yahoo.com.br

**Keywords:** *Lantana montevidensi*, antioxidant activity, fumigant, *Drosophila melanogaster*, biological activity

## Abstract

*Background and objectives:* Natural products such as essential oils with antioxidant potential can reduce the level of oxidative stress and prevent the oxidation of biomolecules. In the present study, we investigated the antioxidant potential of *Lantana montevidensis* leaf essential oil (EOLM) in chemical and biological models using *Drosophila melanogaster*. *Materials and methods:* in addition, the chemical components of the oil were identified and quantified by gas chromatography coupled to mass spectrometry (GC-MS), and the percentage compositions were obtained from electronic integration measurements using flame ionization detection (FID). *Results:* our results demonstrated that EOLM is rich in terpenes with Germacrene-D (31.27%) and β-caryophyllene (28.15%) as the major components. EOLM (0.12–0.48 g/mL) was ineffective in scavenging DPPH radical, and chelating Fe(II), but showed reducing activity at 0.24 g/mL and 0.48 g/mL. In in vivo studies, exposure of *D. melanogaster* to EOLM (0.12–0.48 g/mL) for 5 h resulted in 10% mortality; no change in oxidative stress parameters such as total thiol, non-protein thiol, and malondialdehyde contents, in comparison to control (*p* > 0.05). *Conclusions:* taken together, our results indicate EOLM may not be toxic at the concentrations tested, and thus may not be suitable for the development of new botanical insecticides, such as fumigants or spray-type control agents against *Drosophila melanogaster*.

## 1. Introduction

Reactive species are known to cause damage to cellular membranes (lipid peroxidation), DNA, proteins, and mitochondria, and have been found to be involved in the pathophysiology of a wide range of diseases such as brain ischemia, carcinogenesis, diabetes, etc. [1,2]. Natural products, especially from plant origin, can prevent some of the harmful effects of reactive species [3,4,5,6]. Reports indicate that the consumption of food antioxidants can reduce the incidence of various degenerative diseases, as well as the degenerative process associated with aging [7,8,9]. One of the mechanisms by which these antioxidants exert their beneficial effects is by avoiding the oxidation of biomolecules, which would break the chain reaction of the pathogenesis in the deterioration of the physiological functions [10,11]. 

People’s interest in natural compounds rather than synthetic ones is growing considerably [12], especially because they are regarded as safe with no side effects and possess a variety of therapeutic actions [13]. Indeed, natural products such as vegetable oils with antioxidant potential can help the organism to modify the oxidative state in imbalance conditions [14,15]. In addition, plant essential oils exhibit low mammalian toxicity and can affect the reproduction, growth rate, and behavior of insects [16,17,18,19,20]. *Lantana montevidensis* Briq., popularly known as “chumbinho” in Brazil, is used to treat rheumatism, bronchitis, and gastric disorders [21]. Studies carried out with the leaf extract or leaf essential oil of *L. montevidensis* demonstrated their antibacterial activities and their potential to modulate antibiotics drugs used in clinical infections [22,23,24,25]. In addition, leaf extract of *L. montevidensis* was shown to exhibit antioxidant activity [9], while flavonoids from its leaves were reported to exert antiproliferative activity against gastric adenocarcinoma, human uterus carcinoma, and murine melanoma cells, in vitro [21].

In spite of the fact that numerous studies have reported the insecticidal activity of essential oils against mosquitos and flies, there is a lack of knowledge about the effect of the essential oil from *L. montevidensis* on *Drosophila melanogaster*. Many basic biological, physiological, and neurological properties are conserved between mammals and *D. melanogaster*, and about 75% of the genes causing human diseases have a functional counterpart in the fly [26,27]. Thus, *D. melanogaster* has been widely used as a model in genetic research to study the physiopathology of neurodegenerative diseases such as Parkinson’s disease and Alzheimer’s disease [28,29]. In addition, *D. melanogaster* is accessible to large-scale insecticide screening operations, and it is physiologically, biochemically, and genetically similar to mosquitoes and flies [20,30,31]. Therefore, *D. melanogaster* provides an excellent model system to experimentally evaluate possible fumigant insecticides.

Considering the abovementioned information, the objective of this study was to investigate the chemical characterization and antioxidant potential of *L. montevidensis* leaf essential oil (EOLM) in vitro, as well as its possible toxic effects using *Drosophila melanogaster* as a model. Particularly, we evaluated the toxic effect of the essential oil from *L. montevidensis* on the cell viability (MTT) and on biomarkers of oxidative stress such as lipid peroxidation, iron levels, total thiols, and non-protein thiols (NPSH).

## 2. Materials and Methods

### 2.1. Plant Material

The leaves of *L. montevidensis* were collected in the Medicinal Plants Garden of the Regional University of Cariri—URCA, Crato—CE Brazil (7°22′ S; 39°28′ W and 492 m above sea level), at 9:30 am. After identification, a voucher was deposited in the HCDAL (Herbarium Dárdano de Andrade Lima-URCA) (Crato, Brazil), with number #7518. The leaves were dried in the shade.

### 2.2. Extraction of L. montevidensis Leaf Essential Oil

The dried leaves were crushed and submitted to a hydrodistillation system in a modified Clevenger type apparatus. Three hundred (300) grams of the sample were placed in a 5.0 L glass flask along with 2.0 L of distilled water and heated to boiling for 3 h [32]. The oil was collected using a glass Pasteur pipette, after which the yield was calculated. The obtained essential oil was treated with anhydrous sodium sulphate (Na_2_SO_4_) and stored at −4 °C until the chemical analyzes were carried out. The essential oil showed a yield of 0.19%.

### 2.3. Composition and Identification of the Constituents of the Essential Oil

After obtaining the essential oil, it was submitted to GC/MS analysis according the procedure described in the literature [33].

### 2.4. Reagents

Glutathione (GSH), 1,1,3,3-Tetramethoxypropane (TMP), thiobarbituric acid (TBA), 5,5′-Dithiobis(2-nitrobenzoic acid), (3-(4,5-Dimethylthiazolyl-2)-2,5-diphenyltetrazolium bromide) (MTT), and 1,10-Phenanthroline were purchased from Sigma Aldrich (St. Louis, MO, USA). All the reagents were of analytical grade.

### 2.5. Antioxidant Activity in Chemical Model

#### 2.5.1. Antioxidant Capacity in Chemical Model: 1,1-Diphenyl-2-picrylhydrazyl (DPPH) Radical Scavenging Activity 

The radical scavenging ability of *L. montevidensis* leaf essential oil was performed using the stable free radical DPPH (1,1-Diphenyl-2-picrylhydrazyl) as described by Kamdem et al. [34].

#### 2.5.2. Fe^2+^ Chelating Activity of *L. montevidensis* Leaf Essential Oil 

**Figure 1:**
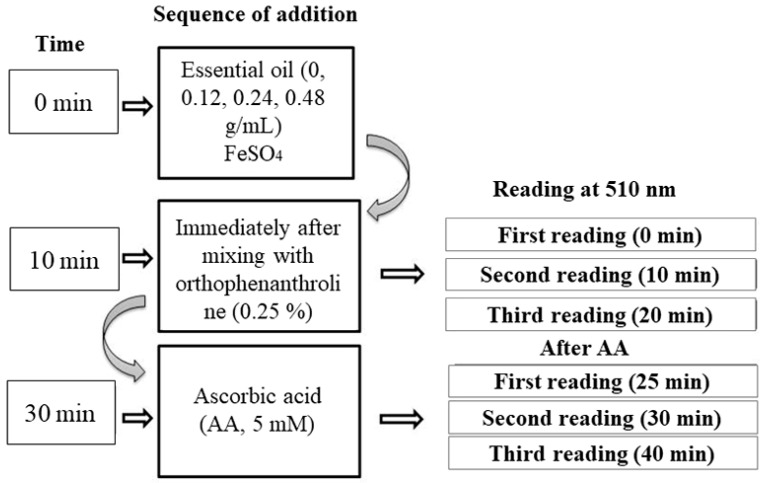
Flowchart of the steps for the assays using Fe^2+^/Fe^3++^ by *L. montevidensis* leaf essential oil.

The chelating capacity of essential oil from the leaves of *L. montevidensis* was determined according to the modified methods of Dinis et al. [35] and Kamdem et al. [36]. To summarize, the schedule for the evaluation of Fe^2+^ chelation or oxidation by the oil is presented in (Figure 1).

#### 2.5.3. Fe^3+^ Reducing Power of *L. montevidensis* Leaf Essential Oil 

To further investigate the reductive potential of the essential oil, the same reaction mixture as described above but using FeCl_3_(110 µM) instead of FeSO_4_(110 µM) in the reaction mixture and was determined using a modified method of Kamdem et al. [36]. 

### 2.6. Biological Assays with Drosophila Melanogaster

#### Fumigant Toxicity

Different concentrations of the essential oil of *L. montevidensis* (EOLM) (0, 0.12, 0.24, and 0.48 g/mL) were prepared by dissolving it in 4% DMSO. The filter paper fragment was treated with 300 μL of different concentrations of EOLM and placed at the bottom of the flask. Then, 50 adult flies (males and females) of 3–5 days were exposed to the oil. The top of the flask was sealed with adhesive foam to prevent evaporation of the essential oil. The control group was exposed to the filter paper soaked with 4% DMSO alone. The flasks were maintained in a light/dark cycle of 12 h, 25 °C ± 1 °C, and 60% of relative humidity. The experiment was performed in triplicate, and the number of death flies was counted after 30 min, 1 h, 2 h, 3 h, 4 h, and 5 h. 

### 2.7. Determination of Total Thiol and Non-Protein Thiols (NPSH)

Twenty flies from each group were homogenized in 1 mL of 0.1 M potassium phosphate buffer, pH 7.4, at a ratio of 1:10 (1 mg of flies for 10 µL), and then centrifuged at 10,000 rpm for 10 min. For the determination of total thiols, 50 μL of the obtained supernatant was added to 190 μL of 0.1 M potassium phosphate buffer (pH 7.4), and then 10 μL of 5 mM DTNB (5,5′-Dithiobis(2-nitrobenzoic acid) was added to the mixture. The reaction mixture was incubated for 30 min at room temperature (protected from light), and the absorbance was measured at 405 nm using microplate reader. Glutathione (GSH) was used as standard, and the results were expressed as ηmol GSH/g of tissue. For the measurement of NPSH level, the obtained supernatant was missed with equal volume of 10% trichloroacetic acid (TCA) and centrifuged for 3 min at 10,000 rpm. The clear supernatant was used for NPSH determination as described for the total thiol. 

### 2.8. Assessment of Cell Viability

Cell viability was assessed with MTT [3-(4,5-Dimethylthiazol, 2-yl)-2,5-diphenyl-2′-tetrazolium bromide], according to the method described by Mosman [37]. A volume of 20 μL of the supernatant from treated and untreated flies was added to 170 μL of potassium phosphate buffer (0.1 M, pH 7.4), followed by the addition of 10 μL of 1 mg/mL MTT prepared in ethanol. The reaction medium was incubated for 120 min, and then 150 μL of the mixture was pipetted and added to 50 μL of DMSO. After 10 min of incubation at room temperature, the readings were carried out at 492 nm and 630 nm, respectively, in an ELISA microplate reader.

### 2.9. Determination of Iron Levels

The iron (II) ions content was determined by measuring the intensity of the orange complexe formed with 1,10-phenanhroline and free Fe^2+^ in the supernatant of control and treated groups with EOLM. The free Fe^2+^ content was determined using a modified method of Kamdem et al. [36] and Klimaczewski et al. [38]. Briefly, ten microliters (10 μL) of 1,10-Phenanthroline (0.25%) was added to the reaction mixture containing 110 μL of saline solution (0.9% NaCl), 60 μL of 0.1 M Tris-HCl (pH 7.4), and 20 μL of the supernatant, and then incubated for 60 min at room temperature. Iron(II) sulfate was used to construct the standard curve. Absorbance was measured after incubation at 492 nm using microplate reader, and the results were expressed in ηmol of Fe (II)/g tissue.

### 2.10. Measurement of Malondialdehyde (MDA)

Thiobarbituric acid reactive substances (TBARS) were measured to determine lipid peroxidation products as a measure of oxidative stress. Ten flies per group, in triplicate, were homogenized and centrifuged at 10,000 rpm for 10 min. Briefly, the reaction mixture containing 100 μL of the supernatant, 100 μL of 10% trichloroacetic acid (TCA), and 100 μL of 0.75% of 2-thiobarbituric acid (TBA, prepared in 0.1 M HCl) was incubated at 95 °C for 1 h. After cooling, they were centrifuged at 10,000 rpm for 10 min, and the absorbance was measured at 405 nm using 250 μL of the reaction mixture [39]. MDA used for the standard curve was obtained by hydrolysis of 1,1,3,3-tetramethoxypropane (TMP). The results were expressed as ηmol MDA (malondialdehyde)/g of tissue.

### 2.11. Statistical Analysis

Results were expressed as mean ± standard error of the mean (SEM). Statistical analysis was performed using one-way (ANOVA), with multiple comparisons of Bonferroni, in order to detect significant differences between controls and treatments, and two-way, for chelating and reducing power. The probability of *p* < 0.05 was considered statistically significant. 

All protocols were approved by the Commission of Ethics in Research in Animals (CEUA) of the Regional University of Cariri (00029/2017.1) on 23 October 2017.

## 3. Results

### 3.1. Chemical Characterization of L. montevidensis Leaf Essential Oil

The essential oil from the dried leaves of *Lantana montevidensis* (EOLM) yielded 0.19%, in which 32 compounds were identified. As it can be seen in Table 1, the chemical composition of EOLM revealed that germacrene D (31.27%), β-caryophyllene (28.15%), biciclogermacrene (6.04%), α-copaene (5.98%), α-humulene (5.81%), and caryophyllene oxide (5.07%) are the major phytochemicals. However, the least represented ones were spathulenol (0.96%), β-elemene (0.84%), t-sabinene hydrate (0.71%), camphor (0.43%), sabinene (0.29%), and camphene (0.11%). 

**Figure 2:**
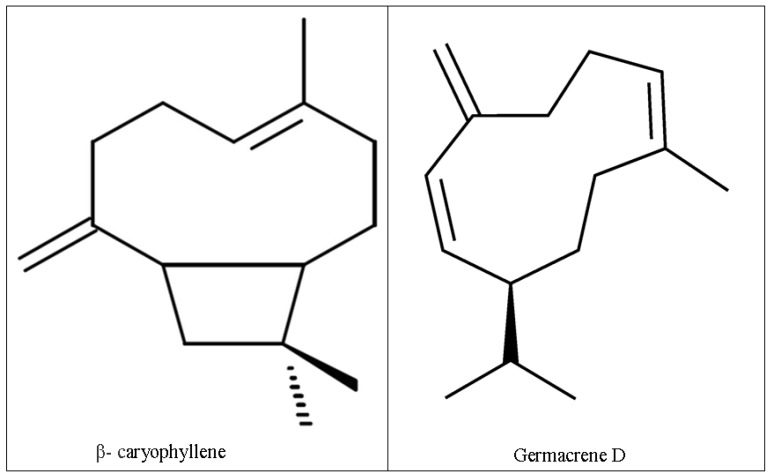
Chemical structures of major compounds in *L. montevidensis* leaf essential oil.

From the results, it is possible to state that sesquiterpenes (e.g., germacrene D and β-caryophyllene) are the major groups of compounds found in EOLM (Table 1, Figure 2). 

### 3.2. Antioxidant Activity

#### 3.2.1. Scavenging Activity of the Essential Oil from *L. montevidensis* on DPPH Radical

**Figure 3:**
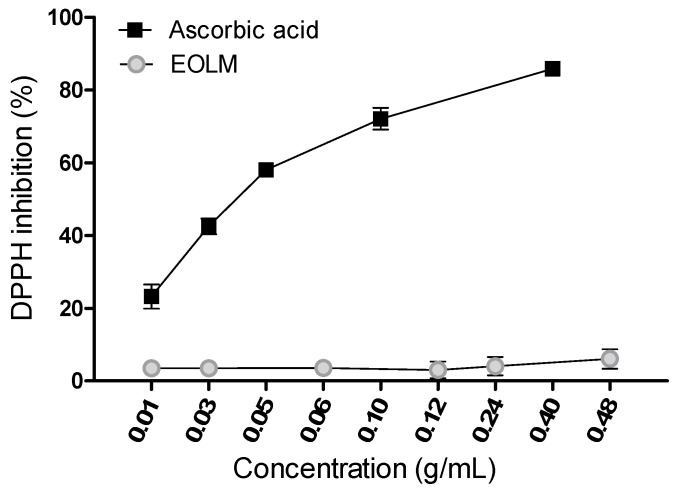
Quenching of 1,1-Diphenyl-2-picrylhydrazyl (DPPH) color by the essential oil from *L. montevidensis*. Mean ± SEM of n = 4 independent experiments.

The effect of essential oil of *L. montevidensis* and ascorbic acid on DPPH reduction is shown in Figure 3. Ascorbic acid exhibited DPPH radical scavenging activity in a concentration-dependent manner (Figure 3), with IC_50_ value of 0.042 g/mL. However, *L. montevidensis* leaf essential oil did not exhibit DPPH radical scavenging activity at all the concentrations tested (Figure 3).

#### 3.2.2. Fe^2+^ Chelation or Oxidation Potential of *L. montevidensis* Leaf Essential Oil

**Figure 4:**
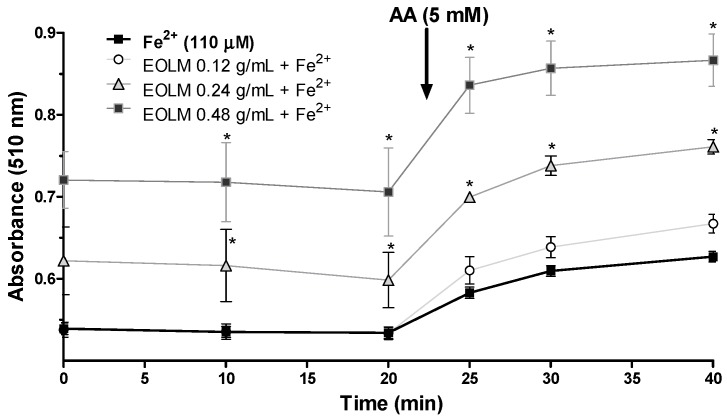
Oxidation of Fe^2+^ by *L. montevidensis* leaf essential oil (0.12–0.48 g/mL). The values represent the mean ± SEM of three experiments that were performed in duplicate.

In Fe^2+^ chelating assay, the rate of the reduction in the absorbance of the orange complex formed by the interaction of Fe^2+^ and ortho-phenanthroline allows estimation of a possible chelator. Surprisingly, the incubation of *L. montevidensis* leaf essential oil (0.24 and 0.48 g/mL) with Fe^2+^ significantly increased the absorbance of the complex Fe^2+^ orthophenanthroline formed, when compared with that of Fe^2+^ alone (Figure 4). The absorbance remained unchanged after 20 min of incubation, but dramatically raised after addition of the reducing agent (ascorbic acid (Figure 4)). This finding may suggest that the leaf essential oil of *L. montevidensis* is oxidizing Fe^2+^ in the reaction medium. 

After 20 min incubation of Fe^2+^ with EOLM, ascorbic acid (AA) was added to the reaction medium to confirm whether or not the increase in absorbance in the presence of the oil was attributed to Fe^2+^. Absorbance did not change at 5, 10, and 20 min after addition of AA, suggesting that EOLM directly stimulated the oxidation of Fe^2+^ to Fe^3+^ during the incubation periods (prior to AA addition) (Figure 4).

#### 3.2.3. Fe3+ Reducing Properties of *L. montevidensis* Leaf Essential Oil

**Figure 5:**
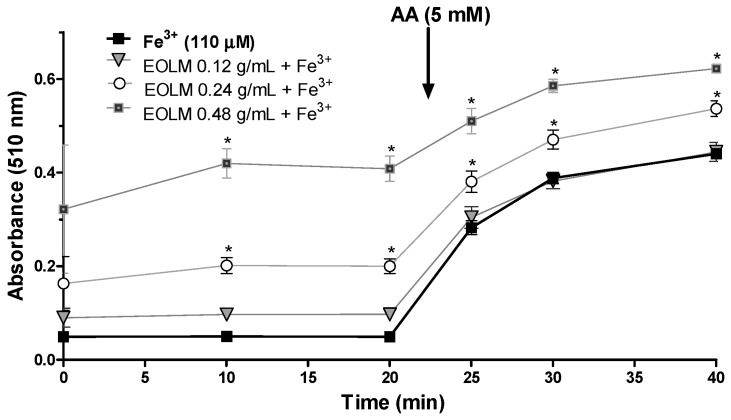
Reduction of Fe^3+^ to Fe^2+^ (110 µM) by *L. montevidensis* leaf essential oil (0.12–0.48 g/mL). The oil was incubated with for 10 min.

The Fe^3+^ reducing properties of *L. montevidensis* is shown in Figure 5. Similar to that observed with Fe^2+^, the incubation of the essential oil of *L. montevidensis* with Fe^3+^ in the presence of ortho-phenanthroline resulted in a significant increase in the absorbance in a dose-dependent manner (*p* < 0.05) in comparison to that of Fe^3+^ alone. However, the absorbance of the complex formed with Fe^3+^ and ortho-phenanthroline was lower than that obtained with Fe^3+^ and ortho-phenanthroline (Figure 5). The addition of ascorbic acid to the reaction medium dramatically increased the absorbance of the sample (Figure 5), suggesting that the component(s) of the essential oil of *L. montevidensis* may have released Fe(II) in the medium or have reduced Fe^3+^ to Fe^2+^. 

### 3.3. Biological Assays with Drosophila Melanogaster

#### 3.3.1. Fumigant Activity of Leaf Essential Oil *L. montevidensis*

The fumigant toxicity of the essential oil extracted from leaves of EOLM was investigated in Drosophila melanogaster. Exposure of flies to EOLM at all the concentrations tested (0.12, 0.24 and 0.48 g/mL) for up to 5 h, caused mortality below 10% (data not shown). 

#### 3.3.2. Quantification of Total Thiol and Non-Protein Thiols (GSH and NPSH)

**Figure 6:**
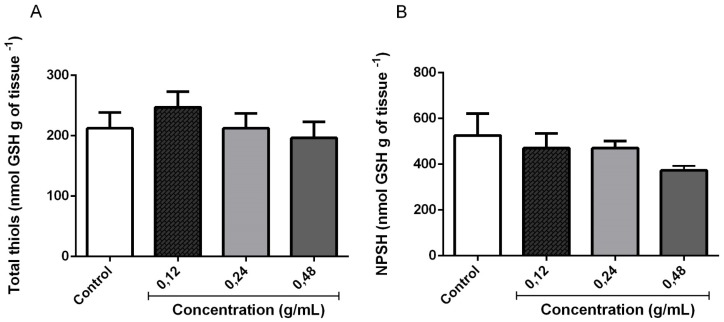
Total thiol (**A**) and non-protein thiol (NPSH) content (**B**) in flies homogenates following 5 h exposure of *D. melanogaster* to *L. montevidensis* leaf essential oil.

Considering that antioxidant activity has been reported for *L. montevidensis* [9], we investigated the effects of EOLM on the indirect biomarker of oxidative stress, total thiol. Additionally, the levels of GSH, an antioxidant found in the intracellular medium, that acts to defend the cell from oxidative stress, were estimated in homogenates of *D. melanogaster*. The results presented in the Figure 6A,B revealed that the treatment with EOLM (0.12–0.48 g/mL) did not significantly alter the levels of total thiol (Figure 6A) and non-protein thiol (NPSH) (Figure 6B), when compared with the control (*p* < 0.05).

#### 3.3.3. Cell Viability of *D. melanogaster* Supernatant

**Figure 7:**
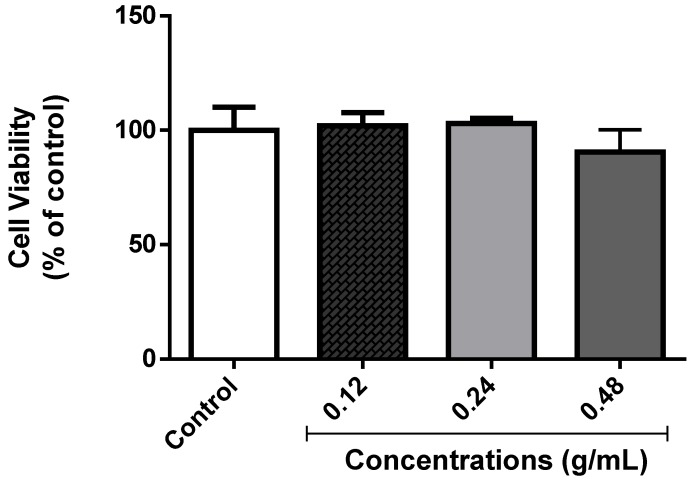
Effect of EOLM on cellular viability of *D. melanogaster* homogenates.

MTT is reduced by the action of the enzyme succinate dehydrogenase in living cells to generate the purple chromophore, which is used to determine cell viability. The formation of the colored product is proportional to the number of viable cells in the supernatant. The EOLM at all concentrations tested did not change cellular viability of the supernatants when compared to the control (Figure 7).

#### 3.3.4. Effect of EOLM on Fe^2+^ Content

**Figure 8:**
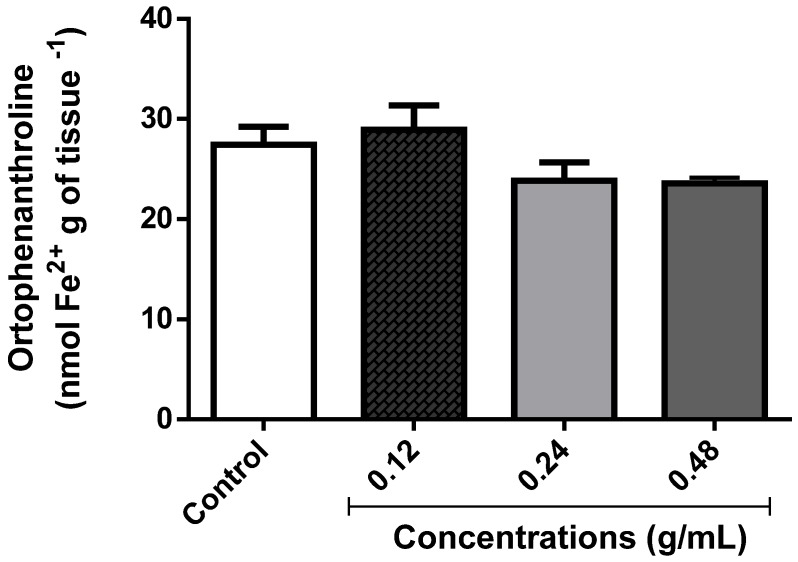
Total iron levels in *D. melanogaster* treated with *Lantana montevidensis* leaf essential oil (EOLM).

To investigate possible oxidative damage, after exposure of *D. melanogaster* to different concentrations of EOLM, the levels of free Fe^2+^ ions were measured. As shown in Figure 8, exposure of flies to EOLM (0.12–0.48 g/mL) did not cause significant change in total iron content compared with the control group (*p* < 0.05).

#### 3.3.5. Determination of Lipid Peroxidation

**Figure 9:**
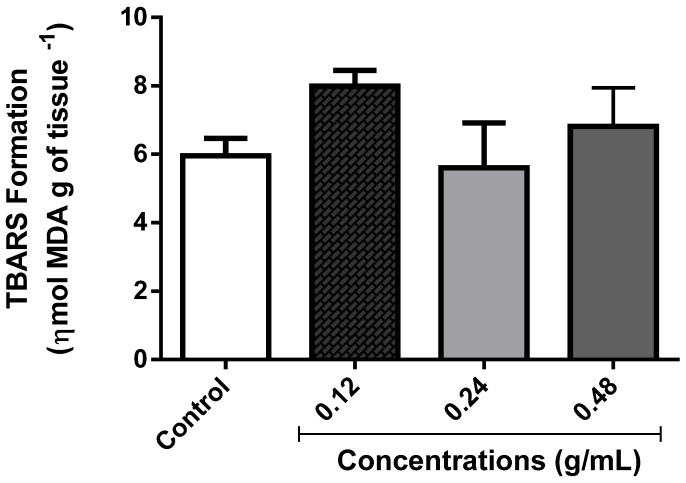
Determination of the content of malondialdehyde (MDA).

MDA, one of the well-known side products of LPO, is used as an index of lipid damage. For this reason, we measured the effect of EOLM on MDA levels following exposure. As shown in Figure 9, the EOLM at different concentrations tested did not alter MDA level in comparison with the control (*p* > 0.05). 

## 4. Discussion

The use of in vivo alternative models in order to perform toxicological tests is increasing [15,40]. In this scenario, several model organisms have been used to identify the pharmacological properties of plants material or active components. *Drosophila melanogaster* is one of them, and has been used for more than 110 years to study the biological effects of compounds and the underlying pathophysiology of numerous diseases, including Alzheimer’s and Parkinson’s diseases [31,41,42].

Medicinal plants have been widely used for the prevention and/or treatment of various diseases, on the basis that they do not present genotoxic risks when consumed for long time [43]. In the current study, the antioxidant activity of *Lantana montevidensis* leaf essential oil was evaluated in vitro, while its potential toxic effect was investigated in vivo using *D. melanogaster*. In general, the essential oil of the leaves of *L. montevidensis* appeared to be rich in terpenes, (mainly, monoterpenes and sesquiterpenes), which can justify its strong odor [44]. EOLM showed only small differences in composition compared to data reported in the literature [24,25]. For instance, in the study carried out by Bezerra et al. [45], the chemical composition of the essential oil of the leaves of *Lantana montevidensis* was β-caryophyllene (34.96%), germacrene D (25.49%), and bicyclogermacrene (9.48%), while in the study by Sousa et al. [24], it was β-caryophyllene (31.5%), germacreno D (27.5%), and bicyclerecyroreno (13.9%). Such differences can be attributed to intrinsic and/or extrinsic factors such as environmental conditions and time and place of leaf collection [46,47,48]. β-caryophyllene (28.15%), one of the major component of *L. montevidensis* leaf essential oil, is a sesquiterpene present in essential oils of several plants families. Plants species that have this constituent exhibit a variety of pharmacological activities such as anti-inflammatory, antinociceptive, and antioxidant, among others [49,50,51]. d-germacrene (31.27%), the major component found in *L. montevidensis* leaf essential oil, is a common compound in plants, being considered a precursor for the biosynthesis of many sesquiterpenes [52]. DPPH is a stable radical, exhibiting a maximum absorbance at 517 nm. Such absorption by the action of antioxidants is taken as a measure of antioxidant activity [53]. It has widespread use in the assessment of radical elimination [54,55]. Here, the essential oil of *L. montevidensis* showed very low DPPH scavenging activity, and Fe(II) chelating activity, but demonstrated higher reducing power. The study carried out by Barros et al. [9] demonstrated the antioxidant activity of the aqueous and ethanolic extracts of *L. montevidensis* against DPPH radical, and its capacity to inhibit lipid peroxidation in rat brain. Our results are in agreement with that of Hossain and Shah [40], who found low antioxidant activity for other essential oils. Consistent with this, essential oils rich in monoterpenes (e.g., from *Cupressus sempervirens*, *Phyllostachys nigra*, *Eucalyptus globulus,* and *Psidium guayava*) have been shown to be ineffective in scavenging DPPH radical [14]. 

Plant essential oils (EOs) have been tested against a wide range of arthropod pests, with promising results. EOs have been reported to possess high efficacy, multiple mechanisms of action, low toxicity in non-target vertebrates, and the potential to act as reducing agents and stabilizers for the synthesis of nanopesticides [56]. Therefore, the reducing power of EOLM observed in this study could justify its potential use as a natural reducing agent.

It is widely recognized that reactive oxygen species (ROS) or reactive nitrogen species (RNS) induce oxidative stress by causing significant damage to cell structure directly or indirectly, leading to a number of diseases [57,58,59]. The major targets of ROS during oxidative stress are thought to be DNA, RNA, proteins, and lipids. Different reactive species have different degrees of reactivity with the cellular components, and the availability of free iron in the form of Fe^2+^ is considered to be of utmost importance in ROS toxicity due to its participation in the Fenton reaction that drives the formation of hydroxyl radicals [60]. Although free Fe^2+^ ions are known to exert vital functions in the organism at low concentrations [61], high level of free Fe^2+^ ions has been detected in several neurodegenerative diseases such as Alzheimer’s disease [62]. Therefore, compounds able to chelate Fe^2+^ to render it unavailable or less available for the participation in the Fenton reaction are of particular interest. Unfortunately, EOLM did not exhibit Fe^2+^ chelating activity.

Glutathione (GSH) is an essential compound in the maintenance of cellular homeostasis because of its reducing properties [63]. Protein thiol groups are inherently very reactive, and can be oxidized by reactive species. The balance between oxidized and free thiols is important not only for the maintenance of protein and enzymatic functions, but also for cellular redox balance [64]. The results obtained in this study showed that EOLM at all the concentrations tested did not alter the total thiol and NPSH levels and the MDA content of flies homogenates, indicating that EOLM does not cause oxidative stress since the total thiol depletion is associated with increased lipid peroxidation [65]. Thus, it is possible to presume that the beneficial effects of EOLM may be attributed at least, in part, to its reducing ability. In spite of the fact exposure of flies to EOLM (0.12–0.48 g/mL) did not affect MDA content (an index of lipid peroxidation), there is report showing the potential of *L. montevidensis* to decrease LPO in egg phospholipid [25], and to exert antioxidant activity in vitro [9,25]. 

## 5. Conclusions

In conclusion, the present study demonstrated that *Lantana montevidensis* leaf essential oil was ineffective in scavenging the DPPH radical, but exhibited reducing activity in vitro. Exposure of *Drosophila melanogaster* to *L. montevidensis* leaf essential oil (0.12–0.48 g/mL) for 5 h did not significantly alter markers of oxidative, as evidenced by there being no change in total thiol, GSH, and MDA contents. 

## Figures and Tables

**Table 1:** Composition of the Lantana montevidensis essential oil.

Compounds	RI ^a^	RI ^b^	Oil Composition (%) *L. montevidensis*
α-Pinene	939	937	nd
Camphene	953	951	0.11
Sabinene	976	675	0.29
β-Pinene	980	983	nd
Myrcene	991	990	nd
α-Terpinene	1018	1015	nd
p-Cymene	1026	1026	nd
(Z)-β-Ocimene	1040	1037	nd
(E)-β-Ocimene	1050	1054	nd
γ-Terpinene	1062	1061	nd
Terpinolene	1088	1079	1.67
*cis*-Linalool oxide	1074	1074	3.86
Linalool	1098	1199	1.09
Camphor	1143	1141	0.43
Terpin-4-ol	1177	1174	nd
α-Terpineol	1189	1193	nd
*t*-Sabinene hydrate	1254	1257	0.71
α-Copaene	1376	1376	5.98
β-Elemene	1391	1389	0.84
β-Caryophyllene	1404	1401	28.15
(E)-Caryophyllene	1418	1423	2.49
Aromandendrene-allo	1461	1460	1.12
α-Humulene	1454	1451	5.81
Germacrene D	1480	1480	31.27
Valencene	1491	1489	nd
Biciclogermacrene	1494	1497	6.04
Cubebol	1514	1518	nd
δ-Cadinene	1513	1509	nd
α-Cadidene	1538	1538	3.84
Spathulenol	1576	1573	0.96
Caryophyllene oxide	1581	1585	5.07
**Total identified (%)**			**99.73**

The relative proportions of the essential oil constituents were expressed as percentages. nd—not determined; ^a^ retention indices of the literature (Adams, 1995). ^b^ Retention of experimental indices (based on homologous series of n-C_7_-C_30_ alkane).

## References

[1] Hua H., Xing F., Selvara J.N., Wang Y., Zhao Y., Zhou L., Liu X., Liu Y. (2014). Inhibitory effect of essential oils on *Aspergillus ochraceus* growth and ochratoxin a production. PLoS ONE.

[2] Kamdem J.P., Abolaji A.O., Elekofehinti O.O., Omotuyi I.O., Ibrahim M., Hassan W., Barbosa N.V., Souza D.O., Rocha J.B.T. (2016). Therapeutic Potential of Plant Extracts and Phytochemicals Against Brain Ischemia-Reperfusion Injury: A Review. Nat. Prod. J..

[3] Fang Y.Z., Yang S., Wu G. (2002). Freeradicals, antioxidants, andnutrition. Nutrition.

[4] Rao K.M., Shashidhara L.S. (2008). Human APC sequesters β-catenin even in the absence of GSK-3β in a *Drosophila* model. Oncogene.

[5] Chikezie P.C., Uwakwe A.A. (2011). Membrane stability of sickle erythrocytes incubated in essential oil of three medicinal plants: Anacardium occidentale, Psidium guajava, and Terminalia catappa. Pharmacogn. Mag..

[6] De Beer D., Joubert E., Gelderblom W.C.A., Manley M. (2017). Phenolic compounds: A review of their possible role as in vivo antioxidants of wine. S. Afr. J. Enol. Vitic..

[7] Bekhit A.E.D.A., Cheng V.J., McConnell M., Zhao J.H., Sedcole R., Harrison R. (2011). Antioxidant activities, sensory and anti-influenza activity of grape skin tea infusion. Food Chem..

[8] Vasilescu I., Eremia S.A., Albu C., Radoi A., Litescu S.C., Radu G.L. (2015). Determination of the antiradical properties of olive oils using an electrochemical method based on DPPH radical. Food Chem..

[9] Barros L.M., Duarte A.E., Waczuk E.P., Roversi K., da Cunha F.A.B., Rolon M., Coronel C., Gomez M.C.V., de Menezes I.R.A., da Costa J.G.M. (2017). Safety assessment and antioxidant activity of *Lantana montevidensis* leaves: Contribution to its phytochemical and pharmacological activity. EXCLI J..

[10] Scarfiotti C., Fabris F., Cestaro B., Giuliani A. (1997). Free radicals, atherosclerosis, ageing, and related dysmetabolic pathologies: Pathological and clinical aspects. Eur. J. Cancer Prev..

[11] Garcia G.R., Goodale B.C., Wiley M.W., La Du J.K., Hendrix D.A., Tanguay R.L. (2017). In vivo characterization of an AHR-dependent long non-coding RNA required for proper Sox9b expression. Mol. Pharmacol..

[12] Carmona-Jiménez Y., García-Moreno M.V., Igartuburu J.M., Barroso C.G. (2014). Simplification of the DPPH assay for estimating the antioxidant activity of wine and wine by-products. Food Chem..

[13] Musa K.H., Abdullah A., Kuswandi B., Hidayat M.A. (2013). A novel high throughput method based on the DPPH dry reagent array for 2 determination of antioxidant activity. Food Chem..

[14] Sacchetti G., Maietti S., Muzzoli M., Scaglianti M., Manfredini S., Radice M., Bruni R. (2005). Comparative evaluation of 11 essential oils of different origin as functional antioxidants, antiradicals and antimicrobials in foods. Food Chem..

[15] Yassa N., Masoomi F., Rankouhi S.R., Hadjiakhoondi A. (2015). Chemical composition and antioxidant activity of the extract and essential oil of Rosa damascena from Iran, population of Guilan. DARU J. Pharm. Sci..

[16] Xie Y.J., Wang K., Huang Q.Y., Lei C.L. (2014). Evaluation toxicity of monoterpenes to subterranean termite, Reticulitermes chinensis Snyder. Ind. Crops Prod..

[17] Neahoua-Bougherra H.H., Bedini S., Cosci F., Flamini G., Belhamel K., Conti B. (2015). Enhancing the insecticidal efficacy of inert dusts against stored food insect pest by the combined action with essential oils. IOBC-WPRS Bull..

[18] Mansoura S.A., El-Sharkawy A.Z., Abdel-Hamid N.A. (2015). Toxicity of essential plant oils, in comparison with conventional insecticides, against the desert locust, *Schistocerca gregaria* (Forskål). Ind. Crops Prod..

[19] Peixoto M.G., Bacci L., Blank A.F., Araújo A.P.A., Alves P.B., Silva J.H.S., Santos A.A., Oliveira A.P., da Costa A.S., Blank M.F.A. (2015). Toxicity and repellency of essential oils of *Lippia alba* chemotypes and their major monoterpenes against stored grain insects. Ind. Crops Prod..

[20] Zhang Z., Yang T., Zhang Y., Wang L., Xie Y. (2016). Fumigant toxicity of monoterpenes against fruitfly, *Drosophila melanogaster*. Ind. Crops Prod..

[21] Nagao T., Abe F., Kinjo J., Okabe H. (2002). Antiproliferative constituents in plants. 10 flavones from the leaves of *Lantana montevidensis* BRIQ. and consideration of structure activity relationship. Biol. Pharm. Bull..

[22] Barreto F.S., Sousa E.O., Rodrigues F.F.G., Costa J.G.M., Campos A.R. (2010). Antibacterial Activity of *Lantana camara* Linn *Lantana montevidensis* Briq extracts from Cariri-Ceara, Brazil. J. Young Pharm..

[23] Sousa E.O., Almeida T.S., Rodrigues F.F., Campos A.R., Lima S.G., Costa J.G. (2011). *Lantana montevidensis* Briq improves the aminoglycoside activity against multiresistant *Escherichia coli* and *Staphylococcus aureus*. Indian J. Pharmacol..

[24] Sousa E.O., Barreto F.S., Rodrigues F.F., Campos A.R., Costa J.G. (2012). Chemical composition of the essential oils of *Lantana camara* L. and *Lantana montevidensis* Briq. and their synergistic antibiotic effects on aminoglycosides. J. Essent. Oil Res..

[25] Sousa E.O., Rodrigues F.F.G., Campos A.R., Lima S.G., da Costa J.G.M. (2013). Chemical composition and synergistic interaction between aminoglycosides antibiotics and essential oil of *Lantana montevidensis* Briq.. Nat. Prod. Res..

[26] Nichols C.D. (2006). *Drosophila melanogaster* neurobiology, neuropharmacology, and how the fly can inform central nervous system drug discovery. Pharmacol. Ther..

[27] Pandey U.B., Nichols C.D. (2011). Human disease models in *Drosophila melanogaster* and the role of the fly in therapeutic drug discovery. Pharmacol. Rev..

[28] Feany M.B., Bender W.W. (2000). A *Drosophila* model of Parkinson’s disease. Nature.

[29] Tiwari S., Gondhalekar A.D., Mann R.S., Scharf M.E., Stelinski L.L. (2011). Characterization of five CYP4 genes from Asian citrus psyllid and their expression levels in *Candidatus* Liberibacter asiaticus-infected and uninfected psyllids. Insect Mol. Biol..

[30] Zolfaghari Emameh R., Syrjänen L., Barker H., Supuran C.T., Parkkila S. (2015). *Drosophila melanogaster*: A model organism for controlling Dipteran vectors and pests. J. Enzyme Inhib. Med. Chem..

[31] Panchal K., Tiwari A.K. (2017). *Drosophila melanogaster* “a potential model organism” for identification of pharmacological properties of plants/plant-derived components. Biomed. Pharmacother..

[32] Matos F.J.A. (2002). Introduction to Experimental Phytochemistry.

[33] Adams R.P. (2007). Identification of Essential Oil Components by Gas Chromatography/Mass Spectrometry.

[34] Kamdem J.P., Stefanello S.T., Boligon A.A., Wagner C., Kade I.J., Pereira R.P., Preste A.S., Roos D.H., Waczuk E.P., Appel A.S. (2012). In vitro antioxidant activity of stem bark of *Trichilia catigua* Adr. Juss. Acta Pharm..

[35] Dinis T.C.P., Madeira V.M.C., Almeida M.L.M. (1994). Action of phenolic derivates (acetoaminophen, salycilate and 5-aminosalycilate) as inhibitors of membrane lipid peroxidation and as peroxyl radical scavengers. Arch. Biochem. Biophys..

[36] Kamdem J.P., Adeniran A., Boligon A.A., Klimaczewsk C.V., Elekofehinti O.O., Hassan W., Ibrahim M., Waczuk E.P., Meinerz D.F., Athayde M.L. (2013). Antioxidant activity, genotoxicity and cytotoxicity evaluation of lemon balm (*Melissa officinalis* L.) ethanolic extract: Its potential role in neuroprotection. Ind. Crops Prod..

[37] Mosmann T. (1983). Rapid colorimetric assay for cellular growth and survival: Application to proliferation and cytotoxicity assays. J. Immunol. Methods.

[38] Klimaczewsk C.V., Saraiva R.A., Roos D.H., Boligon A., Athayde M.L., Kamdem J.P., Barbosa N.V., Rocha J.B.T. (2014). Antioxidant activity of *Peumus boldus* extract and alkaloid boldine against damage induced by Fe (II)–citrate in rat liver mitochondria in vitro. Ind. Crops Prod..

[39] Barbosa Filho V.M., Waczuk E.P., Kamdem J.P., Abolaji A.O., Lacerda S.R., da Costa J.G., de Menezes I.R., Boligon A.A., Athayde M.L., da Rocha J.B. (2014). Phytochemical constituents, antioxidant activity, cytotoxicity and osmotic fragility effects of Caju (*Anacardium microcarpum*). Ind. Crops Prod..

[40] Hossain M.A., Shah M.D. (2015). A study on the total phenols content and antioxidant activity of essential oil and different solvent extracts of endemic plant *Merremia borneensis*. Arab. J. Chem..

[41] Siddique Y.H., Mujtaba S.F., Jyoti S., Naz F. (2013). GC-MS analysis of Eucalyptus citriodora leaf extract and its role on the dietary supplementation in transgenic *Drosophila* model of Parkinson’s disease. Food Chem. Toxicol..

[42] Liu Q.F., Lee J.H., Kim Y.M., Lee S., Hong Y.K., Hwang S., Oh Y., Lee K., Yun H.S., Lee I.S. (2015). In vivo screening of traditional medicinal plants for neuroprotective activity against Aβ42 cytotoxicity by using *Drosophila* models of Alzheimer’s disease. Biol. Pharm. Bull..

[43] Bakkali F., Averbeck S., Averbeck D., Idaomar M. (2008). Biological effects of essential oils—A review. Food Chem. Toxicol..

[44] Guilhon de Castro H., Borges de Moura Perini V., Rodrigues dos Santos G., Castro Alves Barros Leal T. (2010). Avaliação do teor e composição do óleo essencial de *Cymbopogon nardus* (L.) em diferentes épocas de colheita. Revista Ciência Agronômica.

[45] Bezerra J.W.A., Rodrigues F.C., Costa A.R., Boligon A.A., da Rocha J.B.T., Barros L.M. (2017). Estudo químico-biológico do óleo essencial de *Lantana montevidensis* (chumbinho) (Spreng.) Briq. (Verbenaceae) contra *Drosophila melanogaster*. Rev. Bras. Plantas Med..

[46] Charles D.J., Simon J.E. (1990). Comparison of extraction methods for the rapid determination of essential oil content and composition of basil. J. Am. Soc. Hortic. Sci..

[47] Jorge S.S.A., Nardes P.R.B., Guarim N.G., Macedo M. (1998). O uso medicinal da arnica, Brickelia brasiliensis (Spreng.) Robinson (Asteraceae). Revista Saúde e Ambiente.

[48] Facanali R., Campos M.M.S., Pocius O., Ming L.C., Soares-Scott M.D., Marques M.O.M. (2009). Biologia reprodutiva de populações de *Ocimum selloi* Benth. Rev. Bras. Plantas Med..

[49] Passos G.F., Fernandes E.S., da Cunha F.M., Ferreira J., Pianowski L.F., Campos M.M., Calixto J.B. (2007). Anti-inflammatory and anti-allergic properties of the essential oil and active compounds from Cordia verbenacea. J. Ethnopharmacol..

[50] Marinho D.F., Oliveira E.C.P.D., Araújo J.A.D.S., Pinto I.F., Lima H.S.D., Moraes W.P., Ambrósio C.E., Morini A.C. (2017). Evaluation of ultrasonic transmission of Copaifera duckei Dwyer herbal gel. Pesqui. Veterinária Bras..

[51] Martins A.O., Rodrigues L.B., Cesário F.R., de Oliveira M.R., Tintino C.D., e Castro F.F., Alcântara I.S., Fernandes M.N., de Albuquerque T.R., da Silva M.S. (2017). Anti-edematogenic and anti-inflammatory activity of the essential oil from Croton rhamnifolioides leaves and its major constituent 1, 8-cineole (eucalyptol). Biomed. Pharmacother..

[52] Steliopoulos P., Wüst M., Adam K.P., Mosandl A. (2002). Biosynthesis of the sesquiterpene germacrene D in Solidago canadensis: 13C and 2H labeling studies. Phytochemistry.

[53] Frankel E.N., Meyer A.S. (2000). The problem of using one- dimensional methods to evaluate multifunctional food and biological antioxidants. J. Sci. Food Agric..

[54] Siddhuraju P., Becker K. (2003). Antioxidant properties of various solvent extracts of total phenolic constituents from three different agroclimatic origins of drumstick tree (*Moringa oleifera* Lam.) leaves. J. Agric. Food Chem..

[55] Siddhuraju P., Becker K. (2007). The antioxidant and free radical scavenging activities of processed cowpea (*Vignaunguiculata* (L.) Walp.) seed extracts. Food Chem..

[56] Pavela R., Benelli G. (2016). Essential oils as ecofriendly biopesticides? Challenges and constraints. Trends Plant Sci..

[57] Pistón M., Machado I., Branco C.S., Cesio V., Heinzen H., Ribeiro D., Fernandes E., Chisté R.C., Freitas M. (2014). Infusion, decoction and hydroalcoholic extracts of leaves from artichoke (*Cynara cardunculus* L. subsp. cardunculus) are effective scavengers of physiologically relevant ROS and RNS. Food Res. Int..

[58] Gupta D.K., Palma J.M., Corpas F.J. (2015). Reactive Oxygen Species and Oxidative Damage in Plants under Stress.

[59] Wang J., Hu S., Nie S., Yu Q., Xie M. (2016). Reviews on mechanisms of in vitro antioxidant activity of polysaccharides. Oxidative Med. Cell. Longev..

[60] Mittler R. (2017). ROS are good. Trends Plant Sci..

[61] Oliveira F., Rocha S., Fernandes R. (2014). Iron metabolism: from health to disease. J. Clin. Lab. Anal..

[62] Connor J.R., Malecki E.A., Cable E.E., Isom H.C. (2002). The lipophilic iron compound TMH-ferrocene [(3, 5, 5-trimethylhexanoyl) ferrocene] increases iron concentrations, neuronal l-ferritin, and heme oxygenase in brains of BALB/c mice. Biol. Trace Elem. Res..

[63] Rooney J.P. (2007). The role of thiols, dithiols, nutritional factors and interacting ligands in the toxicology of mercury. Toxicology.

[64] Ferreira I.C., Abreu R. (2007). Oxidative stress, antioxidants and phytochemicals. Bioanálise.

[65] Ghorbel I., Khemakhem M., Boudawara O., Marrekchi R., Jamoussi K., Amar R.B., Boudawara T., Zeghal N., Kamoun N.G. (2015). Effects of dietary extra virgin olive oil and its fractions on antioxidant status and DNA damage in the heart of rats co-exposed to aluminum and acrylamide. Food Funct..

